# HIV and Dyadic Intervention: An Interdependence and Communal Coping Analysis

**DOI:** 10.1371/journal.pone.0040661

**Published:** 2012-07-12

**Authors:** Catherine M. Montgomery, Charlotte Watts, Robert Pool

**Affiliations:** 1 Institute for Science, Innovation and Society, School of Anthropology and Museum Ethnography, University of Oxford, Oxford, United Kingdom; 2 Amsterdam Institute for Social Science Research, University of Amsterdam, Amsterdam, The Netherlands; 3 Faculty of Public Health & Policy, London School of Hygiene & Tropical Medicine, London, United Kingdom; 4 Barcelona Centre for International Health Research (CRESIB), University of Barcelona, Barcelona, Spain; 5 Centre for Social Science and Global Health, University of Amsterdam, Amsterdam, The Netherlands; University of California, San Francisco, United States of America

## Abstract

**Background:**

The most common form of HIV transmission in sub-Saharan Africa is heterosexual sex between two partners. While most HIV prevention interventions are aimed at the individual, there is mounting evidence of the feasibility, acceptability, and efficacy of dyadic interventions. However, the mechanisms through which dyadic-level interventions achieve success remain little explored. We address this gap by using Lewis et al’s interdependence model of couple communal coping and behaviour change to analyse data from partners participating in an HIV prevention trial in Uganda and Zambia.

**Methods and Findings:**

We conducted a comparative qualitative study using in-depth interviews. Thirty-three interviews were conducted in total; ten with couples and twenty-three with staff members at the two sites. The Ugandan site recruited a sero-discordant couple cohort and the Zambian site recruited women alone. Spouses’ transformation of motivation is strong where couples are recruited and both partners stand to gain considerably by participating in the research; it is weaker where this is not the case. As such, coping mechanisms differ in the two sites; among sero-discordant couples in Uganda, communal coping is evidenced through joint consent to participate, regular couple counselling and workshops, sharing of HIV test results, and strong spousal support for adherence and retention. By contrast, coping at the Zambian site is predominantly left to the individual woman and occurs against a backdrop of mutual mistrust and male disenfranchisement. We discuss these findings in light of practical and ethical considerations of recruiting couples to HIV research.

**Conclusions:**

We argue for the need to consider the broader context within which behaviour change occurs and propose that future dyadic research be situated within the framework of the ‘risk environment’.

## Introduction

Estimates suggest that between 35% and 93% of new heterosexually-acquired HIV infections in sub-Saharan Africa occur in married or cohabiting couples [1]–[4] and that serodiscordancy is high. For example, reporting on 12 sites from East and Southern Africa, Lingappa et al found that among all couples with one HIV-1 infected partner, almost half (49%) were HIV-1 discordant [5]. Whilst early evidence suggested that directing interventions, such as voluntary counselling and testing, to the couple could have important benefits [6]–[9], the vast majority of sexual health interventions are directed at the individual. In the past two years, couple-focused research has gained momentum, with empirical reviews and theoretical frameworks proliferating [for example, 10,11–21]. In a systematic review of studies testing whether couples-based behavioural interventions reduce HIV transmission and risk behaviour, Burton et al found that “results across studies consistently indicated that couples-focused programs reduced unprotected sexual intercourse and increased condom use compared with control groups” [11]. Likewise, in a review of couple-based HIV prevention in the United States, El-Bassel concluded that “couple-based intervention strategies have been rigorously tested and are a valuable addition to the arsenal of HIV prevention strategies” [13].

Not only have couple-level interventions been demonstrated to be effective (see above), there is also evidence they are feasible, acceptable and cost-effective [22], [23]. Although the challenges to recruiting couple cohorts should not be underestimated, various studies have reported ways to overcome these and the importance of doing so [15], [24], [25]. For example, in a pilot study in rural KwaZulu-Natal, McGrath et al succeeded in recruiting heterosexual couples from the general population, in spite of high levels of migration and non-cohabitation in this population [20]. In a multi-site study of couple-oriented prenatal HIV counselling, Orne-Gliemann and colleagues found high levels of acceptance amongst staff and beneficiaries, in spite of the fact that the intervention challenged established gender norms and hospital practices [21]. These findings are supported by a study by Kebaabetswe et al, who found a preference for couple, as opposed to individual, HIV counselling and testing among stakeholders in Botswana [17].

Nonetheless, while the popularity of couple-based interventions is increasing, the mechanisms through which they lead to beneficial behaviour change remain largely unspecified [13]. A recent call for theoretical work to move beyond individual cognitive-based models [26], such as the Health Belief Model [27] and the Theory of Reasoned Action [28], has led to several new proposals [16], [29]. Karney et al’s ‘Dyadic Framework for Incorporating Dyads in HIV Prevention’ offers a list of variables likely to influence safe sex, and emphasises dyadic interaction as a mediator and moderator of individual and structural level variables [16]. The paper marks important conceptual progress beyond individual-based models, highlighting the importance of distal level determinants, such as cultural context, in determining interpersonal behaviours. However, as the authors themselves observe, whilst the framework identifies and organises the relevant levels of analysis, it does not propose how exactly the variables work together to affect behaviour.

Filling an important gap in this respect is Lewis et al’s [30] interdependence model of couple communal coping and behaviour change. Although this model was not developed specifically for HIV, it offers a series of constructs mapping the mechanisms through which health behaviour change among couples can be understood. Based on interdependence theory and communal coping perspectives, the model identifies interpersonal factors as key to transforming spouses’ motivation to avoid risk behaviours and to act cooperatively in adopting health-enhancing behaviour change. Interdependence, a core concept in dyad-level social psychological theory, refers to the ways in which bilateral influence between interacting partners affects the outcomes (behaviour or experience) of one or both of them [31]–[33]. Transformation of motivation is a construct used to account for changes in couple members’ behaviour from self-centred to relationship-oriented and health-enhancing. It occurs where a partner interprets health events as meaningful for the relationship or their spouse, rather than simply for themselves. In other words, the motivation underlying behaviour change is given a relational explanation, rather than being ascribed to internal, individual factors such as health beliefs or self-efficacy [30].

Lewis et al combine this relational understanding of motivation with the notion of communal coping to explain how couples work together to achieve better health. Drawing on the work of Lyons and colleagues [34], Lewis et al write that “communal coping refers to couple members holding a shared assessment of a health threat and a vision of shared action about managing the event… The process of communal coping involves (a) one or both couple members holding beliefs that joint effort is advantageous, needed or useful; (b) couple members communicating about the situation; and (c) the couple engaging in cooperative action to solve problems” [30]. In their model, initiation and maintenance of health-enhancing behaviours is a function of communal coping, itself dependent on spouses’ transformation of motivation. The latter is influenced by predisposing factors of the couple, such as the partners’ perceptions of the health threat as a cue to action and their communication style.

In this paper, we use Lewis et al’s model to interpret dyadic qualitative data collected from couples participating in a phase III microbicide trial in Zambia and Uganda, as well as interviews with trial staff. Although very few studies have conducted interviews with both partners simultaneously [35]–[39], theoretically, there is a rationale for doing so; as Lewis et al note: “…methods that capture actual discussions of communal coping within couples may best elucidate how couples communicate, discuss, or decide on communal coping approaches or which patterns of interdependence couples choose to pursue when discussing behaviour change to reduce health threats” [30]. Applying the interdependence and communal coping approach, we compare data between couples recruited together as part of an HIV serodiscordant cohort, and couples where only the female partner was recruited, as well as staff experiences of the two designs. In so doing, we provide an empirical basis for understanding the mechanisms through which couple-focused HIV prevention works.

## Methods

### Ethics Statement

Ethics approval was obtained in the UK from the London School of Hygiene & Tropical Medicine Research Ethics Committee, in Uganda from the Uganda Virus Research Institute Science & Ethics Committee and in Zambia from the University of Zambia Research Ethics Committee. All participants gave written informed consent to participate in the study.

Data were collected during the Microbicides Development Programme (MDP) phase III trial of the candidate microbicide PRO 2000. The trial, known as MDP301, was an international, multi-centre, randomised, double-blind, placebo-controlled trial to evaluate the safety and effectiveness of PRO 2000 for the prevention of vaginally acquired HIV infection. The trial ran from October 2005 to August 2009 and recruited a total of 9,385 women at six African research centres. Five of the centres recruited women and a sixth, in Uganda, recruited serodiscordant couples. Details of the trial’s methodology and results have been published elsewhere [40]–[43]. Briefly, women who consented and who had tested HIV negative, were asked to insert a vaginal gel (PRO 2000 or placebo, randomly allocated) within an hour before each act of sexual intercourse. All participants received HIV testing and counselling, promotion of safer sex practices, free condoms and diagnosis and treatment of sexually transmitted infections.

For this study, we interviewed a purposively selected sample of staff and couples participating in the trial at sites in Uganda and Zambia in 2008 and 2009. In Uganda, HIV-serodiscordant couples, in which the man was HIV-positive and the woman HIV-negative, were identified through sero-survey, and asked to participate in the trial. Both partners’ consent was required for the couple to enrol. In Zambia, women only were recruited to the trial, both from the general community and through employment-related healthcare on a sugar estate near the town of Mazabuka. All women were required to be sexually-active in order to enrol in the trial and were all thus involved in sexual partnerships; male partners in Zambia were of unknown serostatus. In this qualitative research, we wanted to explore differences in women’s and men’s experiences of the trial and the behaviour change it occasioned according to whether they had been recruited together as a couple, or as an individual (women only).

Our sample consisted of ten couples participating in MDP301, five each in Uganda and Zambia. Interviewing couples together was designed to shed light on the processes of communication between partners in decision-making regarding trial participation and behaviour change in relation to HIV. Couples were selected in consultation with local social scientists working on the trial, with key criteria being their willingness to be interviewed together and the likelihood of generating rich accounts. Interviews were conducted by CM with English/vernacular (Luganda, Bemba, Nyanja) interpretation provided by a trial social scientist at each centre. Interviews were loosely structured around the following topics: history of the couple’s relationship; daily routine, including division of labour; how they enrolled in the trial; their experience of HIV testing and using gel and condoms; gender and decision-making; changes experienced as a result of trial participation.

Interviews were also conducted with MDP301 trial staff in Zambia and Uganda about their experiences of running the research, and their views on the advantages and disadvantages of working with couples versus individual women. Nine staff members were interviewed in Uganda and fourteen in Zambia from a range of disciplines and seniority. Interviews were conducted in English by CM. All interviews (bar one, where the participant did not consent to the recording) were digitally recorded, transcribed, translated where necessary and imported into NVivo 8.0 for analysis. Thematic content analysis was used to categorize salient themes in respondents’ accounts, with particular attention paid to the dyadic nature of the couple data. A coding scheme was developed using inductively-generated codes from the data as well as core concepts from Lewis et al’s interdependence model of couple communal coping and behaviour change. All transcripts were systematically coded, with coded text then compared to explore similarities and differences in the data. Pseudonyms and professional roles are used to mask study participants’ identity in this paper.

## Results

Although this study was not designed to test Lewis et al’s model or to measure the determinants of health-enhancing behaviours, the data nonetheless exemplify a number of Lewis et al’s propositions. Given space limitations we do not address every aspect of the model and all its indicators, but focus on those elements most salient in our data, namely couple members’ perceptions of health threats; communication; transformation of motivation; and communal coping. The first two of these are classified as ‘predisposing factors of the couple’ in the model, while transformation of motivation and communal coping are envisaged as fundamental drivers of the outcome, initiation and maintenance of health-enhancing behaviours.

### Uganda: Serodiscordant Couples Recruited Together

In Uganda, apart from a small number of sero-negative concordant couples recruited to blind the community to participants’ HIV status, the trial consisted of HIV serodiscordant couples, where the man was HIV positive and the woman negative. Couples were required to share their test results with each other in order to participate in the trial. As such, couple members’ perceptions of the health threats to themselves and their relationship were acute, and clearly demonstrated in the interviews. In accounts of how they came to enrol on the trial, the couple’s knowledge of their discordant status was presented as a major stimulus for eliciting behaviour change; for men, the motivation was twofold: to access treatment for their own health and to preserve their wife’s health for the benefit of their children. For women, the sure knowledge of their partner’s HIV status made sex a life-or-death activity unless they could use a condom or gel. This high level of perceived threat to each partner, to the couple and to the family unit was a recurrent theme; one of the study clinicians remarked on this:

…we are dealing with a special group of people, people in discordant relationships; most men tell me, ‘we’re trying to look for life, we’re trying to look for health. We don’t want to die, we want to sustain ourselves, we want to have someone who’ll take care of the children. Now because we have this other option, other than the condom, we want to use it. We think it is good.’ (Study clinician, Uganda).

Amongst the Ugandan couples interviewed, data suggested that the partners all held a shared assessment of the threat of HIV and committed to shared action regarding how to manage this. The threat itself was multidimensional: for the man already infected with HIV, the threat was that his wife would leave him, he would have no one to care for him when he got sick, and his offspring might be abandoned if he infected his wife and she also succumbed to the disease. For the seronegative wife, the primary threat was that of HIV infection itself. The process of communal coping occurred on the following basis:

- Both couple members believed that joint effort was needed and to their advantage: the basis of this was acceptance of their serodiscordant status
- They communicated about the situation: this was facilitated by trial staff who provided ongoing individual and couple counselling
- They engaged in cooperative action to solve problems: including use of condoms and gel

Managing the threat of HIV became a joint problem and a joint responsibility for the couple. The recruitment process was central to this, since both partners felt equally targeted by the intervention and were addressed together as part of a dyadic unit. Accounts of the decision to join the research uniformly referred to the process as a joint endeavour that followed mutual discussion, as illustrated here:

Interviewer: How did you come to participate in this study?

Edward: It was in the year 2005 or 2003, when that organization MRC was sent to us and they came to our village here….

Nafuna: …and they drew blood*….*

Edward: …and they drew blood. When they took the blood, we did not immediately get to know our results. There is a health worker that came back and brought our results and interpreted them to us and then went away. But he counselled us on how we can protect ourselves. And then later they came back and told us that we were needed in Masaka.

Nafuna: They came back, is it to Masaka that we went?

Edward: Yes, to Masaka.

Nafuna: We went to Masaka and started getting seminars.

Edward: Seminars. (Couple 7, Uganda).

In this account, the dialogue itself enacts a form of togetherness, with each spouse contributing to the story and echoing the other’s words.

As part of recruitment, informed consent played an important role in formalising a particular version of health behaviour. In Uganda, where both spouses were required to give consent, it concretised a vision of joint action and communal coping. Only where both partners were committed to this, were they enrolled:

…here, it’s an inclusion criteria that the man and the woman agree to participate, so if one of them is still hesitant and needs time, we’ve had counselors actually saying, ‘this couple needs time’ and they’ve been left…Because here…we are really enrolling them as couples. (Social scientist, Uganda).

Ongoing couple counselling and seminars provided by the trial to groups of couples were also key conduits for dyadic communication, a core facilitator of communal coping. These sessions not only delivered study information, but emphasised key behavioural expectations such as faithfulness, unity and open communication.

Edward: In the seminars at first they taught us what was most important, and they told us to remain faithful and united. The issue about faithfulness was there in all the seminars. It is what is important, it was like the theme to be faithful to each other so that we are able to use that gel. (Couple 7, Uganda).

Keeping these people together in their relationship, the study has offered a contribution in terms of the counseling. The counseling itself that they are given - we encourage them to stay together, we encourage them to respect each other, we encourage them to help each other. (Community mobiliser, Uganda).

The seminars and counselling created a space for both partners to confront their discordancy together and to discuss their emotional and behavioural responses to it. Communication between spouses was predominantly reported to occur in the presence of research staff, but this did not necessarily extend back into the home. For example, several couples said that there was no need to discuss the use of the gel every time they used it, since they had both already discussed its use with the researchers. Its use was routine. Crucially, the research *intervened* in couples’ communication norms, forcing a dialogue about safe sex and particularly the use of condoms. Because the couple was the unit of interest, intervention into patterns of communication occurred between both partners:

Peter: To me the relationship has become better because we got that sensitization on using condoms. Before, they used to say that we should use condoms, and she was also told, but we had never got the chance to be together to know why a condom should be used. We went together (to MDP) so that we were able to be told face to face… And usually it is hard for one to express oneself comfortably to a partner, but when you get someone else to intervene…you feel the barriers being removed. (Couple 8, Uganda).

Couple HIV testing and joint seminars were a key factor in spouses’ (especially male spouses’) transformation of motivation in Uganda. As suggested above, joint testing and knowledge of serodiscordancy was key to acceptance of behaviour change in terms of using gel and condoms. An HIV negative woman whose partner did not accept to use gel and condoms could leave him and return to her parents, leaving him to cope with his illness on his own. For him, there would be the additional threat of infecting his wife and the two of them dying early, abandoning their children. Individual knowledge of one’s status would not have secured this transformation of motivation, since either partner could choose to ignore the behavioural implications of unprotected sex. But in sharing their results, jointly attending counselling and both giving written informed consent to participate in the study, the partners committed to a shared goal of behavioural intervention, as this staff member suggests:

I mean the woman knows what it is all about, the man knows what it is all about and the fact that they agree to share these things - it empowers the woman to know, ‘OK, if I’m using this product, I’m really using it to make sure that this happens. I’m using a condom to make sure this happens’. The man also says, ‘No, I have to use this condom because I don’t want my wife to die, we have children’. There’s a kind of responsibility that comes on…a sense of empowerment that really comes in to support these two people. (Social scientist, Uganda).

Couple counselling featured prominently in accounts not only of recruitment to the study and adherence to condom and gel use, but also in terms of retention. Staff reported being vigilant to problems arising between couples, and acting quickly to address these where they occurred, through home visits and further counselling sessions. Therefore, although the quality of the couple relationship itself was not specified as an outcome in the trial, the relationship became a key focus of intervention in the daily life of the research.

Counsellor: It’s mainly separation and death, that’s what affects retention here.

Interviewer: Do you have a chance, when a couple is considering dropping out, to intervene and counsel them before that happens?

Counsellor: Yah we do. When we detect it, women report it, or at times men report it, that, ‘my wife has changed’ - whenever such reports occur, we follow-up. And we put avenues in place to detect such cases. Mobilisers go out and they meet these volunteers, so they are able, in their interactions with the volunteers, to detect family problems… Nurses also have home visits they make, so whenever they detect an anomaly in a relationship…counsellors are supposed (to go), whenever there is need to go and talk to these people, to see how much they can be helped and the relevant problems at hand. (Counsellor, Uganda).

### Zambia: Seronegative Women Recruited Alone

In Zambia, recruitment drives were directed to local women, and only women were eligible to enrol in the trial. In interviews, women demonstrated an acute perception of their own risk of contracting HIV from their primary partner and frequently presented this as their primary reason for attending the research centre in the first place. In the couple interviews there was little effort to disguise this fact in front of their partners, as the following extracts demonstrate:

Grace:…as you know, these Zambian men, they don’t stick to one sexual partner…he can be married to one woman and have four or five girlfriends. So if you’re not careful, you can die together with the girlfriends, so it’s better you protect yourself (Couple 1, Zambia).

Interviewer: What made you join the programme?

Mary: What made me is the gel. As you know, men, like he said, he had a girlfriend. So I heard that maybe gel, if at all it works, it can save my life. (Couple 2, Zambia).

Their partners, by contrast, were much less likely to discuss their own vulnerability to HIV or other sexually transmitted infections. Although some men volunteered the information that they had tested for HIV, knowing their status was not a prerequisite for their partner’s trial participation. The social acceptance of multiple partners for men did not apply to women, so once they knew their wife was negative, they presumed themselves to be negative too: there was no additional benefit to them in using condoms or the experimental gel.

In Zambia, couple counselling was not standard and men rarely came to the research site. Interactions took place between the researchers and individual women, or occasionally, between researchers and individual men. Although women were strongly encouraged to disclose their participation and their use of study product to their partners, it was up to them to find a way to do this.

Interviewer: How did you manage to convince him (about the study)?

Grace: Ah, it’s easy…You convince a man when you are clever [all laugh]…with a word of love, you see, you tell him…then because he loves you, he will follow. But if you just do this with a command, he can’t (agree), unless you go softly.

John: So as you can see, you (the woman) have to suggest it…Sometimes we agree, but it takes time, it takes courage, yes. It did take courage for her to convince me. I didn’t even want it, for sure. But now I’m here and I’m doing it, I think we will come to the end of it… (Couple 1, Zambia).

While many women successfully negotiated their participation in the trial, staff gave numerous reports of women who failed to convince their partners or who had tried to avoid telling them all together. In some such cases, women were said to have been stopped from participating by their partners, or physically beaten when they discovered gel use.

The effect of having a third party present in spousal discussions of condom use, as described by some of the Uganda couples (above), was enacted in the same interview with John and Grace, who had been together for eight years. It became apparent during the interview that John was not fully conversant with his partner’s view on condoms:

Grace: It is easier to use gel than condoms, but for us women, we’d prefer using condoms than gel, but….

John: Is it so?

Grace: Yah.

John: Sure?

Grace: Eh.

John: [Disbelieving] No! [Laughs] How can someone say…You know, gel and condoms, you’re saying it’s better to use condoms than gel? Ah, I don’t think…you’re a woman anyway, I think you’re lying to me. But for me, a man, I don’t like a condom, I think gel is better, especially if she does it without me knowing…I think I would like that rather than a condom.

Interviewer: You wouldn’t mind if she inserted gel without telling you?

John: I wouldn’t mind. But anyway, this time, because I know that she does use it, but what I’m saying is that whether I would have known or not known, gel is good.

Grace: It’s better.

John: And it’s better in fact, it’s better than a condom. Otherwise…because…if you didn’t know or didn’t see her inserting gel you may not know that there is gel here. But for a condom, whether I didn’t see her, I will still know there is a condom. Especially if she’s using her (female) condom - “no, no, there is a condom here!”.

Interviewer: [To Grace] But women would prefer condoms?

Grace: [Hesitating] Mmm…partly, but for us we got gel as the best, yah, it’s better than a condom. The reason why I said condoms is because you remain as you are, you see, just after he releases, it (sperm) will remain in the condom, you are clean [John laughs]. That’s why I like it (the condom). But for men [chuckling], they like gel, in fact, even for both of us we liked gel (more) than condoms and he enjoys, I also enjoy using gel… (Couple 1, Zambia).

In this interview, John seemed to be in genuine disbelief that Grace would prefer a condom. In the extract, they are talking about pleasure, but she is also talking in undertones about sexual safety. She cleverly uses the word ‘clean’ to encompass both a physical sensation of cleanliness – the sperm stay in the condom – and a physical state of safety – she remains virus-free. The exchange is playful, but belies a more serious topic of discussion between the partners about fidelity and trust. When the difference in their views about condoms becomes apparent, Grace effects some discursive work to harmonise their position, emphasizing that gel is best and subsuming her individual opinion into a joint preference for gel: “in fact, even for both of us, we liked gel (more) than condoms”. While this interaction provides an interesting example for the purposes of this study, couples did not routinely attend the trial site together, and therefore did not routinely have the opportunity for such interactions. By contrast, in Uganda, couple counselling provided just such a forum for dyadic interaction about sexual health.

Whereas in Uganda, the couples’ serodiscordant status was discussed as a key stimulus for communal coping and health-enhancing behaviour, in Zambia, transformation of motivation was much less evident. For the man, there was, at least initially, no gain to be had from supporting his partner to participate in the trial, as illustrated below:

Interviewer: So do you think that this MDP programme will benefit you in future?

Julius: I wouldn’t know.

Interviewer: Why do you say so?

Julius: I don’t know in future, MDP are the ones who would know everything. Because right now I wouldn’t just accept that maybe in future I would find the benefits.

….

Interviewer: So do you think that men are very involved in the MDP programme?

Julius: Women are the ones that are very involved in the programme; men - there is nothing.

Interviewer: Why do you say so?

Julius: Because men do not use gel, it’s women who use gel. (Couple 3, Zambia).

Lack of male motivation, and in some cases disenfranchisement, ran as a common theme through the couple and staff interviews, with men often said to feel left out of the research. The strategy of recruiting women without their partners granted agency to individual women, but in many cases – and in all of the couples interviewed – resulted in women seeking permission from their partners to enrol in the study. Rather than men buying into the intervention, then, or claiming a stake in it, their role remained that of gatekeeper to women’s actions.

Interviewer: When she told you that, ‘I want to join the programme for gel’, what did you think about it?

Julius: I allowed her…because she had already come here and she even told me, we even agreed at home. At home, when a woman comes and tells you, ‘big man, where I am going it is like this and that’…she is supposed to ask for permission from me, the man, you see. So me, the man, when I allow her, then everything is just clear. (Couple 3, Zambia).

Verity: …in most cases there is no way you can hide from a man…you cannot say, ‘I should be doing it alone,’ because for the man, that’s his house. What about the day he will discover those things; what will you say? That time it will be bad for you…he will beat you up. When he sees them (the gels) he will beat you and chase you, ‘you go to your (parents’) home. (Couple 5, Zambia).

As these extracts illustrate, communication in the Zambian context occurred when permission was being sought by the woman for her behaviour and, as suggested by some, against the tacit threat of conflict if they did not. Accounts demonstrated attempts by women to engage their partners and instigate communal coping, but this was a struggle where no transformation of motivation had occurred on their spouses’ part. Although some men did accompany their wives on occasion to the research centre, reminded them to insert the gel, or supported them in other ways, there was no sustained incentive for them to do so.

Just as the informed consent process played an important role in formalising a particular version of health behaviour in Uganda, so in Zambia also. Here, the informed consent process enacted the Western liberal aspiration of individual autonomy, in line with the hope that microbicides would be a woman-controlled technology. Nonetheless, signalling the importance of both partners to the outcome of the trial, one of the research staff responded:

Men are very important in such kinds of programme, because for women to participate they need the go-ahead from their partners. Without the go-ahead from the partners, then how is she going to use it? When that person comes in through the door, the husband will ask ‘what’s that? Take it back!’ She’s going to throw it away. I’ve heard of some who are hiding gel by the neighbour’s, in the bush, I don’t know, but they hide it. But how do they use it at 01∶00? When the husband demands for sex at 01∶00, how is she going to use it? (Study clinician, Zambia).

His words underscore the fundamentally dyadic nature of sexual behaviour and the potential tensions that arose for women enrolling as individuals (see also [44], [45]).

## Discussion

Our analysis suggests that recruiting the couple to HIV prevention interventions may play an important role in catalysing mechanisms through which behaviour change is initiated and maintained, namely dyadic transformation of motivation and communal coping. In our research, we have pioneered the use of qualitative dyadic data to advance understanding of the mechanisms through which dyadic-level HIV interventions may achieve success.

Serodiscordancy plays an important part in these findings, which gives this paper particular salience, due to the high prevalence of HIV discordance among married or cohabiting partners in Africa [46], [47]. For example, using DHS data from five African countries, de Walque et al found that at least two thirds of infected couples were discordant [48]; Allen has also characterised discordant couples as ‘Africa’s largest HIV at-risk group’ [49]. Bearing this in mind, our findings underscore the large, and largely untapped, potential of couple voluntary counselling and testing as a prevention tool in these populations, already advocated by other authors [1], [6], [8], [50].

There are, however, caveats to consider. Firstly, this study was not designed to measure the psychosocial determinants of behaviour change, nor was our sample designed to be representative. Site-level differences, such as the long-standing presence of the Medical Research Council at the Uganda site and the lack of previous intervention research at the Zambian site, may have contributed to the findings. We have used Lewis et al’s model post-hoc as a way to guide analysis and interpretation of the data, rather than setting out a priori to test the model. Rigorous empirical validation of the model is required to expand on these preliminary findings. Nonetheless, the data provide important comparative insights into the mechanisms through which couple-level intervention may achieve gains in the initiation and maintenance of health-enhancing behaviours over and above interventions directed at one partner alone.

Secondly, it could also be argued that because the Ugandan men had already been recruited into the trial and counselled and tested, they were different in a fundamental way to the Zambian men, and that the sampling was therefore biased. However, there is no reason to assume that the Ugandan men recruited through the sero-survey were in any way different with regard to the issues discussed in this paper from the Zambian men who had not been recruited and tested. Our data strongly suggest that it was recruitment, and the concomitant changes that this brought about in knowledge and communication within couples, that initiated change and resulted in the difference. In order to test this in a more generalizable way a larger study would need to be conducted in the same population. Although it is something that is broadly recognised in constructivist approaches in social science, this study provides additional evidence that the factors that determine changes in attitudes and behaviour are complex and that the studies that we carry out to study change themselves affect the behaviour we are trying to study. Although there is no way of eliminating such influences entirely, being aware of the possibility that research itself can act as an intervention does help us take this into account when interpreting results.

While Lewis et al do not focus on the context within which behaviour change occurs, our data also suggest the salience of this to the success of the intervention. Context is key to understanding how dyadic behaviour change operates in different cultures, since it does not always occur, as the model assumes, in a context of mutual joint control over behaviour [51]. In both Uganda and Zambia, control over behaviour is strongly skewed in favour of the male spouse. An extensive literature documents the need to consider the power relations between men and women that impact on health-seeking behaviour [see for example 52]. Indeed, vaginal microbicides were developed precisely in response to women’s lack of control over sexual decision-making [53]. We therefore follow Bloor et al’s [54] articulation of risk behaviour as “a situated product, emergent from the immediate situation of the sexual encounter”, and, beyond that, suggest our findings be considered not in isolation, but as part of the social embodiment of the risk environment [55].

The risk environment has been defined as “the space, whether social or physical, in which a variety of factors exogenous to the individual interact to increase vulnerability to HIV” [56]. Work exploring the social structural production of risk has been in part a response to the deficits of individual cognitive-based approaches described in the introduction. It therefore tends away from the immediacy of interpersonal relations to focus on political-economic factors. Yet, key to the risk environment approach is a belief in the inseparability of micro, meso and macro level factors: “structurally determined inequalities find their expression in the micro-social environment and in patterns of individual and community risk behaviour” [56]. It is in this light that we suggest the dyadic environment be given more serious consideration – as the physical site in which HIV transmission occurs; the social site in which communication and decision-making occurs, as well as gender and sexual behaviour norms (re-)produced; the economic site in which male and female partners negotiate co-dependent livelihoods; and the policy site in which laws (including common laws) govern the rights of men and women in family institutions, such as marriage.

In practical terms, consideration needs to be given not only to the potential gains, but also to the challenges of implementing dyadic intervention research. Importantly, there is an ethical question concerning the provision of opportunity to *individuals* to participate in research and interventions when their partners do not wish to do so. This is particularly salient where women are in a position of inequality vis-à-vis their partners. In addition, any benefit of focusing on couples has to be offset against the practical challenges and costs of recruiting them. Recruiting couples to studies is reported to be problematic, entailing logistical difficulties, increased costs, potential for partner coercion and selection bias [25], [57], [58]. However, as we have suggested, the gains may be substantial in terms of both study-related outcomes (such as adherence and retention) and the broader success of efforts to prevent HIV transmission.

Indeed, beyond the question of individual trials, the question is whether we can afford to ignore the dyad any longer in HIV prevention. In a recent study of PrEP adherence in sero-discordant couples in Uganda, Ware et al [59] found that relationship dynamics had a major impact on adherence. Their findings support our illustration that motivation to change sexual behaviour occurs in a relational context in which both partners have a stake, particularly where one partner is HIV positive and the other negative. Programmes that fail to take account of the couple as a unit, and the risk environment in which they reside, miss opportunities for successful risk reduction in both women and men. Rhodes persuasively argues that “environments exhibit relations of risk *and* enablement, of disadvantage *and* capacity” [55]. Through the high-leverage process of communal coping, and as part of the risk environment, dyadic intervention offers the prospect of transforming the primary partnership from a risk factor for HIV infection to a protective resource.
